# Microbial production of *cis,cis*-muconic acid from aromatic compounds in engineered *Pseudomonas*

**DOI:** 10.1016/j.synbio.2023.08.001

**Published:** 2023-08-09

**Authors:** Siyang He, Weiwei Wang, Weidong Wang, Haiyang Hu, Ping Xu, Hongzhi Tang

**Affiliations:** a State Key Laboratory of Microbial Metabolism, and School of Life Sciences & Biotechnology, Shanghai Jiao Tong University, Shanghai, 200240, People's Republic of China; b College of Life Science, Northeast Forestry University, Harbin, 150040, China

**Keywords:** Biodegradation, Polycyclic aromatic hydrocarbons, Biological funneling, *cis,cis*-muconic acid, *Pseudomonas*

## Abstract

Industrial expansion has led to environmental pollution by xenobiotic compounds like polycyclic aromatic hydrocarbons and monoaromatic hydrocarbons. *Pseudomonas* spp. have broad metabolic potential for degrading aromatic compounds. The objective of this study was to develop a “biological funneling” strategy based on genetic modification to convert complex aromatic compounds into *cis,cis*-muconate (ccMA) using *Pseudomonas putida* B6-2 and *P. brassicacearum* MPDS as biocatalysts. The engineered strains B6-2 (B6-2ΔcatBΔsalC) and MPDS (MPDSΔsalC(pUCP18k-catA)) thrived with biphenyl or naphthalene as the sole carbon source and produced ccMA, attaining molar conversions of 95.3% (ccMA/biphenyl) and 100% (ccMA/naphthalene). Under mixed substrates, B6-2ΔcatBΔsalC grew on biphenyl as a carbon source and transformed ccMA from non-growth substrates benzoate or salicylate to obtain higher product concentration. Inserting exogenous clusters like *bedDC*_*1*_*C*_*2*_*AB* and *xylCMAB* allowed B6-2 recombinant strains to convert benzene and toluene to ccMA. In mixed substrates, constructed consortia of engineered strains B6-2 and MPDS specialized in catabolism of biphenyl and naphthalene; the highest molar conversion rate of ccMA from mixed substrates was 85.2% when B6-2ΔcatBΔsalC was added after 24 h of MPDSΔsalC(pUCP18k-catA) incubation with biphenyl and naphthalene. This study provides worthwhile insights into efficient production of ccMA from aromatic hydrocarbons by reusing complex pollutants.

## 1 Introduction

In the last decade, global economies have proliferated and living standards have risen along with surging industrial expansion. This expansion has entailed synthesis and emissions of large volumes of biochemical materials, especially artificial organic compounds. These xenobiotics include polycyclic aromatic hydrocarbons (PAHs) and monocyclic aromatic hydrocarbons (MAHs), which are ubiquitous in atmospheric, aquatic, and terrestrial ecosystems. PAHs and MAHs have cytotoxic, genotoxic, mutagenic, and carcinogenic activities, requiring effective cleanup or removal strategies [1].

Ecological remediation of PAH or MAH contaminated sites faces challenges of hydrophobicity, adsorption, and persistence of pollutants. Bioremediation methods are cost-effective, *in-situ*, and eco-friendly, and can be used to treat these contaminants. There are commonalities in metabolic approaches of microbes that can degrade benzene rings in aromatic compounds. Mono- or di-oxygenases in the upper pathway of degradation allow aromatic hydrocarbons to evolve into aromatic ring-containing but structureless central intermediate compounds (e.g. catechol) through oxidation reactions. In the lower pathway, aromatic compounds undergo dearomatization reactions and enter the tricarboxylic acid (TCA) cycle through ring-opening at the *o-*, *meso-* and *para-*positions under the effect of cyclo-cleavage enzymes [[2], [3], [4]]. A commonality in metabolism facilitates integrated degradation metabolic pathways of microorganisms and transforms pollutants into high-value platform compounds like *cis,cis*-muconate (ccMA).

ccMA, a C6 platform compound with broad use prospects, can undergo a variety of actions as building block chemicals including adipic acid, terephthalic acid (TPA), and trimellitic acid. Such products have high application value in the manufacture of nylon 66, polytrimethylene terephthalate (PTT), polyethylene terephthalate (PET), industrial plastics, resins, pharmaceuticals, and food ingredients [[5], [6], [7], [8], [9]]. In particular, when 1,4-butadiene, a building block for synthetic rubbers, is produced from ccMA in a ferulate decarboxylase mutant, the global market of ccMA alone is estimated to be over USD 100 million [5,7,[10], [11], [12]]. Given its versatility in superior performing bioproducts, ccMA has been identified by Shanks and Keeling as a “bio-privileged” molecule [13]. Current technology is mainly focused on driving natural intermediate metabolites in the shikimate pathway to produce ccMA from glucose. One study employed the β-ketoadipic acid pathway to generate ccMA via the *ortho* cleavage of catechol [8].

In the 1990s, ccMA production through microbial fermentation was common in several industrial chassis strains, such as *Escherichia coli*, *Pseudomonas putida*, *Corynebacterium glutamicum*, and *Saccharomyces cerevisiae*. Microbial fermentation of ccMA is classified into three categories from the viewpoint of feedstock: (i) petroleum-based products, such as benzoate and toluene [14,15]; (ii) food-based carbon sources, glucose, xylose, and glycerol [16]; and (iii) lignocellulose bio-based products, such as caffeic acid, coumaric acid, 4-hydroxybenzoic acid, phenol, syringic acid, and benzoate [17,18]. Recent studies have exploited cellulose derived glucose to produce ccMA [19] reducing the focus on petroleum-based, especially aromatic, contaminant substrates. Due to the occurrence of various compounds in organic contamination, the conveyance of diverse aromatic hydrocarbons to specific chemicals is a pivotal step in the exhaustive handling and development of contaminant utilization. But these compounds are non-renewable, and the reaction conditions are harsh, energy-intensive, and environmentally polluting. Because of this, selecting good chassis strains for utilizing aromatic compounds by biological methods has become a widespread approach [20].

The Gram-negative strain *P. putida* KT2440 and related strains have emerged as candidates for ccMA bioproduction [9]. For instance, inactivation of muconate cycloisomerase (CatB) enabled accumulation of ccMA via the β-ketoadipiate pathway from benzoate [21] and guaiacol [22]; coexpression of PCA 3,4-dioxygenase and PCA decarboxylase allowed ccMA production from softwood lignin without glucose [23]; and silencing of PcaHG and CatBC allowed KT2440-CJ103 to convert *p*-coumarate to ccMA [17]. *P. putida* B6-2 is a metabolically versatile strain that can metabolize aromatic compounds, such as biphenyl fluorine, carbazole, and dibenzofuran. Gene clusters encoding metabolic pathways for central aromatic compounds (benzoate, 4-hydroxybenzoate, protocatechuate, salicylate, and catechol) have been identified in the genome of strain B6-2 [24,25]. *P. brassicacearum* MPDS is a high-efficiency strain utilizing naphthalene, fluorene, and dibenzothiophene as sole carbon sources in cell growth [26], and degrading carbazole, phenanthrene, pyrene, and 2-bromonaphthalene in resting cells [27]. Genomic and transcriptomic analyses identified a naphthalene-degrading gene cluster named *nahAFBCED* with a unique pathway relying on lateral deoxygenation to degrade the aromatics mentioned above. Both strains B6-2 and MPDS isolated from soil contaminated with PAHs and petrochemicals are capable of resisting organic solvents [[26], [27], [28], [29]]. Thus, B6-2 and MPDS can be engineered as potential candidate biocatalysts under extremely harsh conditions to convert PAHs or MAHs into ccMA.

In this study we aimed to develop a stable metabolic engineering strategy to convert aromatic pollutants into ccMA *via* “biological funneling”. We first focused on and channeled the carbon flow of aromatic hydrocarbons toward ccMA synthesis based on the β-ketoadipic acid pathway. ccMA accumulation was achieved with B6-2 (in biphenyl) and MPDS (in naphthalene) without additional carbon sources. We further exploited co-metabolism to allow the conversion of unconvertible substrates. Exogenous metabolic clusters were introduced to broaden the range of substances that could be bio-transformed into ccMA. We also investigated the degradation of composite PAHs and the production of ccMA with the assistance of previously constructed metabolic strains.

## 2 Materials and methods

### 2.1 Strains, chemicals and culture media

*P. putida* B6-2 was obtained from FEM lab [29] and served as the parent strain in this study and the genetic source of *catA*. *P. brassicacearum* MPDS served as the parent strain in this study [26]. *E. coli* TOP10 was used for pUCP18k maintenance and subclone. *E. coli* S17-1 was used for pK18mob-SacB maintenance and the conjugative donor. Biphenyl (≥99.5%, purity), benzoate (≥99.5%, purity) and toluene (≥99.5%, purity) were purchased from Sinopharm Chemical Reagent Co., Ltd. Naphthalene (99% purity) was purchased from J&K Scientific Technology Co., Ltd. Salicylate (99.5% purity) and benzene (≥99.5%, purity) were purchased from Aladdin Bio-Chem Technology Co., Ltd. Seed cultures of *Pseudomonas* spp. and *E. coli* strains were cultivated in Luria-Bertani (LB) broth at 30 °C and 37 °C respectively and supplemented with 100 μg/ml ampicillin (Amp) or/and 50 μg/ml kanamycin (Kan), as propriate. For ccMA bio-production, shake flasks were cultured at 30 °C in the mineral salt medium (MSM) or phosphate buffered saline (PBS) supplemented with suitable antibiotics. For chassis modification, LBS medium and M9 medium with citrate were utilized to screen strains with the correct traits. All kinds of culture media mentioned above were previously described [30], and the solid media were prepared from liquid mediums supplemented with 1.5% agar.

### 2.2 Plasmid construction

Genes were PCR amplified with primers synthesized by Saiheng Biotechnology Co., Ltd. (Table S1). Amplified linear DNA fragments were purified using the TIANgel Purification (TIANGEN). Exogenous metabolic clusters were synthesized by Generay Biotech (Shanghai). ClonExpress Ultra One Step Cloning Kit (Vazyme) was used for plasmid construction, followed by transformation into chemically competent *E. coli* TOP10 or *E. coli* S17-1. Transformants were selected on LB agar (Lennox) plates supplemented with 50 μg/mL kanamycin (Sigma-Aldrich) and grown overnight at 37 °C.

The plasmids used in this study were pK18mob-SacB, pUCP18k and the derivates including pK18-ΔcatB, pK18-ΔsalC, pUCP18k-catA, pUCP18k-bed and pUCP18k-xyl (Table S2). The upstream and downstream of genes like *kkk_4751* (*catB*) or *kkk_2432* (*salC*)were amplified from the chromosome of *P. putida* B6-2, whose purifications were linked for the homologous arms of both by overlapping PCR. Fusion fragment and extracted suicide plasmid were recovered after 6 h of *Eco*RI and *Bam*HI digestion at 37 °C. The fragment and linear vector were blended in a 5:1 ratio and ligated overnight at 16 °C by T4 ligase. The ligated product was transformed into *E. coli* S17-1 by heat shock and positive transformants were screened, which contained pK18-ΔcatB or pK18-ΔsalC. The synthesized cluster *xylCMAB* and *bedDC*_*1*_*C*_*2*_*AB* were codon optimized and straightly integrated into the backbone pUCP18k (Fig. S4, Table S3). The expression plasmid pUCP18k-catA was constructed by In-Fusion clone [31]. The *catA* from *P. putida* B6-2 and the vector pUCP18k was linearized by PCR or inverse PCR with a 15-bp overlap. The products were incorporated at 50 °C for 5 min and cooled down immediately on ice, which was transformed into *E. coli* TOP10.

### 2.3 Strain construction

Gene deletions and replacements were performed with a modified version of the Marx method [32,33]: using the kanamycin-resistant gene as a selection marker for the first round homologous recombination of the plasmid into the chromosome and the sucrose-sensitive gene *sacB* as the counterselection marker for the second round of homologous recombination out of the chromosome. Exogenous cluster expression was only based on the kanamycin selection. Correct gene deletions and exogenous cluster expression were identified by diagnostic colony PCR product based on differences in product size or presence. Plasmids were transformed into *Pseudomonas* by either conjugation or electroporation.

For conjugation, plasmids were built based on backbone plasmid pK18mobSacB, which were transformed from *E. coli* S17-1 (the donor) to *Pseudomonas* strains (the recipient). The overnight cells of strains were cultivated with the same status. Donor and recipient cells were mixed with a ratio of 1:3, resuspended with normal saline and dropped onto an LB plate. Conjugated cells were spread on an M9 salt citrate plate with 100 μg/mL kanamycin for single colonies. And single colonies on the plate were inoculated into LB medium with 100 μg/mL ampicillin and 50 μg/mL kanamycin and verified *via* PCR. The correct single colonies were re-spread on an LBS plate with 100 μg/mL ampicillin and cultivated to select for transconjugants [27]. After the validation by PCR, the knockout strains for B6-2 (B6-2ΔcatB and B6-2ΔcatBΔsalC) and MPDS (MPDSΔsalC) were conclusively constructed (Table S2).

For electroporation [30,34], electrocompetent cells were prepared by centrifuging, washing and resuspending cells in prechilled 10% glycerine. Plasmids for electroporation were constructed within the backbone plasmid pUCP18k and electroporated in a 2 mm cuvette using Gene Pulser Xcell (Bio-Rad) with the preset of 2.5 kV, 25 μF, 200 Ω. All processes mentioned above were performed on ice. The mixture with 1 ml SOC medium was heat-stimulated in a water bath at 46 °C for 6 min and then incubated at 30 °C for 1 h to restore cell activity. The final selection of kanamycin and verification *via* PCR resulted in the individual construction of B6-2ΔcatBΔsalC(pUCP18k-xyl), B6-2ΔcatBΔsalC(pUCP18k-bed), MPDS(pUCP18k-catA) and MPDSΔsalC(pUCP18k-catA) (Table S2).

### 2.4 Degradation and conversion of aromatic compounds

Aromatic compounds’ degradation and conversion were composed of the growth system and resting cell. In the growth system, *Pseudomonas* spp. strains were cultivated in an LB medium with appropriate antibiotics at 30 °C overnight as the seed culture. The flasks were filled with 1% seed culture accompanied by MSM medium and 5 mM of aromatic compounds and cultivated at 30 °C for sampling. Samples were extracted with a triple volume of ethanol after detecting optical density at 600 nm. Overnight seed cultures used in resting cells were cultivated in LB medium with corresponding substrates as inducers under growth conditions, which were added to a liter of LB medium with appropriate antibiotics and induced by the addition of isopropyl β-d-1-thiogalactopyranoside (IPTG) at a final concentration of 0.2 mM upon reaching an optical density at 600 nm (OD_600_) of 0.6. Cells were collected by centrifugation at 4000 rpm for 20 min, whose pellets were rinsed three times with PBS before being resuspended to a final OD_600_ of 5.0 in PBS. The ratio in the mixture of resting cells was considered OD_600_ of 5.0 as the minimum value of 1. The cell suspension was subsequently incubated with 5 mM aromatic compounds at 30 °C, 200 rpm. Samples were periodically drawn, extracted with a triple volume of ethanol, and centrifuged at 12,000 rpm for 5 min to acquire supernatant for metabolite quantification *via* HPLC. Conversion rates were calculated by taking the concentration of ccMA and dividing that by the initial concentration of substrate during the same period.

### 2.5 Fed-batch fermentation

To test ccMA production capability from the probable application scenarios, the modified strains were incubated with industrial PAHs (Fig. S8). Strain B6-2ΔcatBΔsalC was incubated in shake flasks containing MSM medium dosed with 5 mM biphenyl, and MPDSΔsalC(pUCP18k-catA) was incubated in MSM medium with 5 mM naphthalene and 0.2 mM IPTG. The incubation of seed cultures was performed at 30 °C and 200 rpm. For the fed-batch fermentation, 5 mM of corresponding substrates was added to the medium per day. All the fermentation processes were initiated by adding 5 mM of substrate and 1% seed culture. During the fermentation period, samples were harvested to determine the production of ccMA by HPLC. The cell growth was determined with OD_600_.

### 2.6 HPLC metabolite analytical methods

ccMA and each substrate were analyzed with the standard of them to accomplish accurate quantitation (Fig. S3). Separation of each substrate and accumulation of ccMA were achieved using Agilent 1200 series HPLC (Agilent Technologies) coupled with a diode array detector (DAD) and a revered phase C18 column (150 mm × 4.6 mm × 5 μm; Hewlett Packard) after filtration. An injection volume of 10 μL was injected into the column in which the temperature was held constant at 30 °C. The monitored and quantified wavelengths were 230 nm (for benzoate), 254 nm (for biphenyl and ccMA), 275 nm (for naphthalene) and 291 nm (for salicylate). A 1:5 ratio of 0.1% (vol.) formic acid plus water (A) and methanol (B) was utilized at a flow rate of 0.5 mL/min.

## 3 Results

### 3.1 Design of genetic modification strategy

**Fig. 1:**
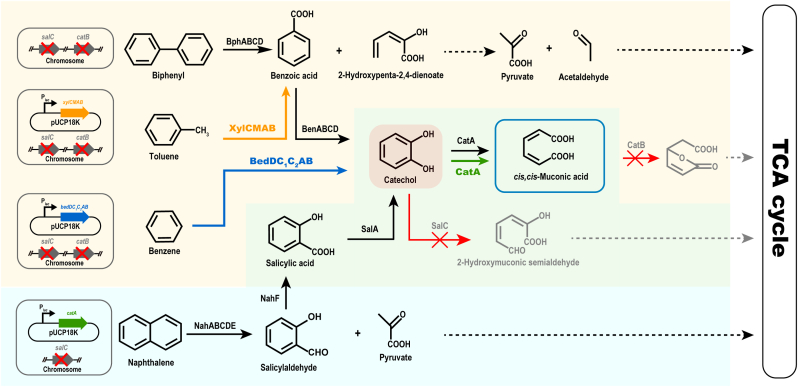
A basic strategy to funnel a variety of aromatic compounds into ccMA biosynthesis in engineered *P. putida* B6-2 and *P. brassicacearum* MPDS. BphA, biphenyl 2,3-dioxygenase; BphB, *cis*-2,3-dihydro-2,3-dihydroxybiphenyl dehydrogenase; BphC, 2,3-dihydroxybiphenyl 1,2-dioxygenase; BphD, 2,6-dioxo-6-phenylhexa-3-enoate hydrolase; XylC, benzaldehyde dehydrogenase; XylMA, xylene monooxygenase & electron transfer component; XylB, benzyl alcohol dehydrogenase; BenABC, benzoate 1,2-dioxygenase; BenD, 1,6-dihydroxycyclohexa-2,4-diene-1-carboxylate dehydrogenase; BedC_1_C_2_AB, benzene dioxygenase; BedD, chlorobenzene dihydrodiol dehydrogenase; CatA, catechol 1,2-dioxygenase; CatB, muconate cycloisomerase; SalA, salicylate hydroxylase; SalC, catechol 2,3-dioxygenase; NahA, naphthalene dioxygenase; NahB, *cis*-naphthalene dihydrodiol dehydrogenase; NahC, 2-dihydronaphthalene dioxygenase; NahD, 2-hydroxy-*2H*-chromene-2-carboxylate isomerase; NahE, *trans*-*o*-hydroxybenzylidenepyruvic hydratase-aldolase; NahF, salicylaldehyde dehydrogenase. To produce ccMA from aromatic pollutants, *salC* and *catB* were inactivated via homologous recombination, and *bed* and *xyl* clusters were introduced by plasmid pUCP18k.

*Pseudomonas* spp. degrade PAHs and heterocyclic derivatives with oxygenase, peroxidase, and dehydrogenase, using catechol as the immediate ccMA precursor in all alternative pathways (Fig. 1). Aromatic ring oxidation by oxygenase is hydroxylated and dehydrogenated to form a central, intermediate catechol. Hence, we structured our biological funnel with catechol as the core intermediate. Modifying the metabolic pathway allowed the aromatic compounds to pool to catechol directly or indirectly (as salicylate or benzoate) during degradation, allowing further conversion to ccMA.

Biphenyl and naphthalene are commonly occurring low molecular weight PAHs. *P. putida* B6-2 has been shown to degrade biphenyl via the β-ketoadipate pathway [24,25,29]. One ring of biphenyl is delivered into the TCA cycle and the other is converted to ccMA from catechol by catechol 1,2-dioxygenase and finally sent to the citric acid cycle. Lack of expression of mucofuranate cycloisomerase (CatB) allowed strain B6-2ΔcatB to accumulate ccMA. Knockout of *salC* enabled B6-2ΔcatBΔsalC to catabolize intermediate catechol by *ortho* cleavage only, ultimately increasing the yield of ccMA from biphenyl. This basic strategy of genetic engineering is also suitable for strain *P. brassicacearum* MPDS.

Strain MPDS utilizes naphthalene as the sole carbon source, transforming it to salicylate by redox, with further catabolism by salicylate-1-hydroxylase and eventual channeling to the central carbon pathway. Through previous genomic analysis [26,27], we found that MPDS itself was *catA* and *catB* deficient and had *salC* encoding catechol 2,3-dioxygenase. Thus, we transformed pUCP18k-catA and pK18-ΔsalC into MPDS so the derivate MPDSΔsalC(pUCP18k-catA) could convert catechol into ccMA *via ortho* cleavage rather than using the *meta* pathway. In response to the superior aromatic degradation of *Pseudomonas*, we attempted to achieve equal degradation and conversion to the target metabolite ccMA by a further expansion of the substrates available to this strain. Introduced with plasmids incorporating the exogenous gene cluster such as *xylCMAB* (*xyl*) and *bedDC*_*1*_*C*_*2*_*AB* (*bed*), mutants B6-2ΔcatBΔsalC(pUCP18k-xyl) and B6-2ΔcatBΔsalC(pUCP18k-bed) were involved in biosynthesis of ccMA from toluene and benzene. This functionality was reliant on the strain's catabolic intermediate—benzoate and catechol in the β-ketoadipic acid pathway.

### 3.2 Strain modification to accumulate ccMA from aromatic compounds

**Fig. 2:**
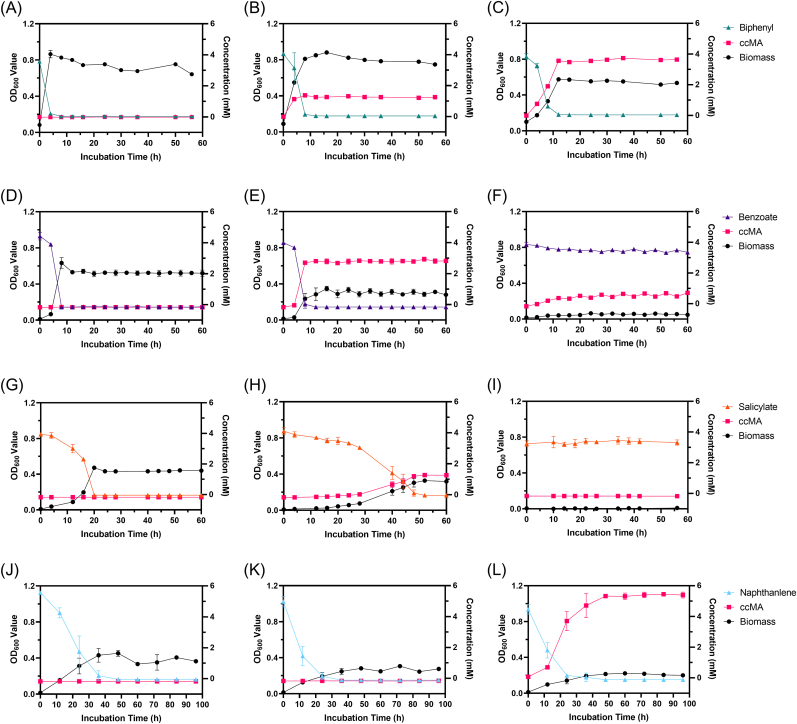
Growth of engineered strains and their ccMA production using different carbon sources. (A) B6-2 wild-type in biphenyl. (B) B6-2ΔcatB in biphenyl. (C) B6-2ΔcatBΔsalC in biphenyl. (D) B6-2 wild-type in benzoate. (E) B6-2ΔcatB in benzoate. (F) B6-2ΔcatBΔsalC in benzoate. (G) B6-2 wild-type in salicylate. (H) B6-2ΔcatB in salicylate. (I) B6-2ΔcatBΔsalC in salicylate. (J) MPDS wild-type in naphthalene. (K) MPDSΔsalC(pUCP18k) in naphthalene. (L) MPDSΔsalC(pUCP18k-catA) in naphthalene. Symbols: filled circles (black), OD_600_; filled squares (red), ccMA; filled triangles (green), biphenyl; filled triangles (purple), benzoate; filled triangles (orange), salicylate; filled triangles (blue), naphthalene. B6-2 wild-type was used in (A), (D) and (G). B6-2ΔcatB was used in (B), (E) and (H). B6-2ΔcatBΔsalC was used in (C), (F) and (I). Initial concentration of each substrate is 5 mM. Each value shown is the means of the results of a triplicate experiment. Error bars indicate standard deviations.

We constructed several genetically engineered microorganisms (GEMs), comprising the CatB-deficient type B6-2ΔcatB, the double-knockout type B6-2ΔcatBΔsalC, the CatA-inserted type MPDS(pUCP18k-catA), MPDSΔsalC(pUCP18k-catA), and other strains. All strains tested, ranging from wild type to single and double knockouts, demonstrated a concomitant decrease in growth activity as the number of knockouts increased (Fig. 2). Based on growth curves in each substrate, the lowest substrate concentration at which strains exhibited significant growth was 5 mM (Fig. S6). Because the sole carbon sources of the two strains were mutually exclusive, a substrate concentration of 5 mM was selected to facilitate subsequent experiments. Under culture conditions with 5 mM biphenyl as the sole carbon source, the OD_600_ value of the B6-2 wild type reached 0.9 after 4 h of incubation, while the *catB*-deficient type only approached 0.9 after 12 h and plateaued. But the double-knockout strain had an OD_600_ of 0.57, much lower activity than the wild-type and *catB* deficient strains.

In cultures of 5 mM salicylate or benzoate, both the wild-type and single-knockout strains grew but the double-knockout strain was inactive. In the benzoate (5 mM) culture, the wild type plateaued after 8 h and the single knockout variant plateaued after 12 h, with the former OD value over 0.6 and the latter only about 0.3. In the salicylate culture, OD_600_ of the wild type peaked at 0.47 at 20 h, while the single knockout strain peaked at 0.33 only after 52 h. For MPDS modified with a similar strategy, growth without *catB* in naphthalene (5 mM) plateaued at 48 h and OD_600_ only reached 0.2 (Fig. 2K and L), but all MPDS strains with *catB* reached 0.4 by 36 h and plateaued (Fig. 2J, S5). The modification strategy was directed at the metabolism and conversion of the target substrate to ccMA by GEMs. Strain B6-2 and its derivates grew with biphenyl, but B6-2 wild type was unable to produce ccMA. B6-2ΔcatB generated 1.4 mM ccMA from 5 mM biphenyl after 8 h of cultivation, while B6-2ΔcatBΔsalC produced 3.7 mM ccMA with nearly 100% yield (mol ccMA/mol biphenyl) after 12 h (Fig. 2A–C, Table 1). This strategy also allowed the engineered *P. brassicacearum* MPDSΔsalC(pUCP18k-catA) to produce ccMA, accumulating approximately 5.4 mM (Fig. 2J-L, S5). But neither B6-2 nor B6-2ΔcatBΔsalC could manufacture ccMA from benzoate and salicylate, while strain B6-2ΔcatB produced 2.9 mM ccMA from 5 mM benzoate after 8 h and 1.3 mM ccMA from salicylate after 52 h (Fig. 2E and H).

**Table 1:** The conversion productivity [mg/(L·h)] and yield rate (mol/mol) of ccMA with different substrates.

Substrate(s)	B6-2	B6-2ΔcatB	B6-2ΔcatBΔsalC	MPDS	MPDSΔsalC(pUCP18k-catA)
Biphenyl	N.D.^a^	24.9, 30.9%	44.3, 95.3%	N.A.^b^	N.A.
Benzoate	N.D.	51.5, 70.5%	1.7, 21.2%	N.A.	N.A.
Salicylate	N.D.	3.6, 30.8%	N.D.	N.A.	N.A.
Biphenyl + Benzoate	N.D.	21.7, 23.6%	16.0, 75.3%	N.A.	N.A.
Biphenyl + Salicylate	N.D.	16.4, 46.2%	12.5, 89.8%	N.A.	N.A.
Naphthalene	N.A.	N.A.	N.A.	N.D.	16.0, 100%
Biphenyl + Naphthalene^c^	N.A.	N.A.	3.3, 11.1%	N.A.	14.1, 46.5%

a N.D. represent “Not Detected”.

b N.A. represent “Not Applied”.

c Only detected in resting cell.

### 3.3 Co-metabolism to produce ccMA from monocyclic aromatic hydrocarbons

**Fig. 3:**
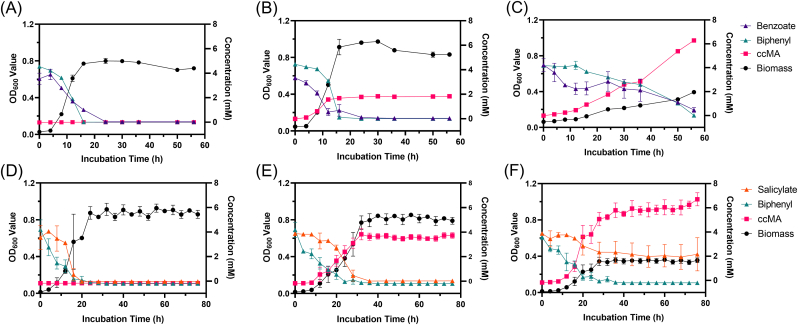
Growth of engineered strains and their ccMA production using combined substrates. (A) B6-2 wild-type in biphenyl and benzoate. (B) B6-2ΔcatB in biphenyl and benzoate. (C) B6-2ΔcatBΔsalC in biphenyl and benzoate. (D) B6-2 wild-type in biphenyl and salicylate. (E) B6-2ΔcatB in biphenyl and salicylate. (F) B6-2ΔcatBΔsalC in biphenyl and salicylate. Symbols: filled circles (black), OD_600_; filled squares (red), ccMA; filled triangles (green), biphenyl; filled triangles (purple), benzoate; filled triangles (orange), salicylate; filled triangles (blue), naphthalene. B6-2 wild-type was used in (A) and (D). B6-2ΔcatB was used in (B) and (E). B6-2ΔcatBΔsalC was used in (C) and (F). Initial concentration of each substrate is 5 mM. Each value shown is the means of the results of a triplicate experiment. Error bars indicate standard deviations.

As described above, B6-2ΔcatBΔsalC grew on biphenyl, but not on salicylate or benzoate. Those MAHs, as non-growth substrates, could be utilized in the presence of a growth substrate like biphenyl. Consequently, the mixture of salicylate (5 mM) or benzoate (5 mM) and biphenyl (5 mM) was tested as the carbon source. In the coculture of benzoate and biphenyl, the wild type reached OD_600nm_ of 0.8 after 16 h of growth, while the single knockout reached 0.9 and the double knockout reached 0.4 at 56 h (Fig. 3). When salicylate was used as one of the substrates, the wild type hit OD_600nm_ of 0.9 after 24 h and plateaued, the single knockout strain reached 0.8 at 32 h and the double knockout strain reached 0.35 at 28 h.

Throughout these experiments, biphenyl was preferentially degraded by strains to produce ccMA and promote the degradation of non-growing substrates for transformation. When mixed with benzoate, biphenyl was entirely degraded by B6-2 and B6-2ΔcatB in about 16 h, 8 h faster than the complete mineralization of benzoate, and B6-2ΔcatBΔsalC consumed biphenyl until 56 h with 0.5 mM of benzoate remaining (Fig. 3A–C). For biphenyl mixed with salicylate, B6-2 and its mutants decomposed biphenyl between 24 h and 28 h. Excepting strain B6-2ΔcatBΔsalC, which left nearly 2 mM of salicylate in the medium, degradation of salicylate by the other two was almost synchronized with biphenyl (Fig. 3D–E). At the final time-point of the experiment, strain B6-2ΔcatBΔsalC produced 6.3 mM ccMA or 6.7 mM ccMA with higher than 75% yield [mol ccMA/(mol benzoate or salicylate plus biphenyl)] (Table 1). Compared to B6-2ΔcatBΔsalC, strain B6-2ΔcatB produced 3.7 mM ccMA from salicylate mixed with biphenyl and 1.83 mM ccMA from a mixture of benzoate and biphenyl.

### 3.4 Insertion of exogenous gene clusters

**Fig. 4:**
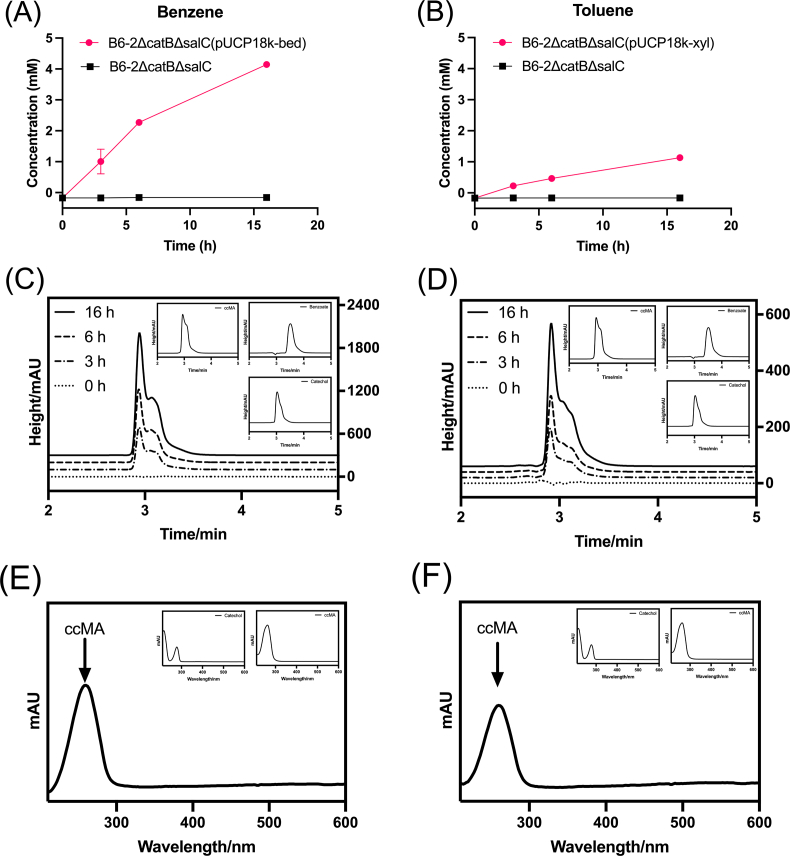
ccMA accumulation in the resting cell of engineering bacterium. (A) B6-2ΔcatBΔsalC(pUCP18k-bed) in benzene. (B) B6-2ΔcatBΔsalC(pUCP18k-xyl) in toluene. (C) ccMA chromatogram of B6-2ΔcatBΔsalC(pUCP18k-bed). (D) ccMA chromatogram of B6-2ΔcatBΔsalC(pUCP18k-xyl). (E) Full wavelength scanning of B6-2ΔcatBΔsalC(pUCP18k-bed) samples. (F) Full wavelength scanning of B6-2ΔcatBΔsalC(pUCP18k-xyl) samples. Symbols: filled circles (red), treatment group including B6-2ΔcatBΔsalC(pUCP18k-bed) and B6-2ΔcatBΔsalC(pUCP18k-xyl); filled squares (black), control group B6-2ΔcatBΔsalC. Initial concentration of each substrate is 5 mM. Each value shown is the means of the results of a triplicate experiment. Error bars indicate standard deviations.

Gene cluster *bedDC*_*1*_*C*_*2*_*AB* encodes a benzene dioxygenase, and *xylCMAB* is responsible for dehydrogenation, methylation, and monoxidation of toluene or xylene. Their introgression gives the deficient B6-2 the ability to degrade benzene or toluene and xylene. Resting cells of B6-2ΔcatBΔsalC(pUCP18k-bed) and B6-2ΔcatBΔsalC(pUCP18k-xyl) were thus utilized to detect the yield of ccMA in the flask with 5 mM benzene or toluene. Dual knockout strain B6-2ΔcatBΔsalC introduced into the cluster *bed* or *xyl* progressively utilized benzene or xylene and subsequently produced the target compound ccMA. After 16 h of reaction, the former GEM yielded 4.1 mM ccMA with a conversion rate of 82%, while the latter produced 1.1 mM ccMA at a rate of only 22% (Fig. 4A and B). Compared with the chromatograms of standard products, no signals of benzoate or catechol were detected during the accumulation of ccMA (Fig. 4C–F).

### 3.5 Degradation of composite PAHs by microbial consortia

Genetically modified strains B6-2 and MPDS were co-cultured to decompose the equal biphenyl-naphthalene mixed substrate. In the growth system, biphenyl (5 mM) and naphthalene (5 mM) were entirely digested, and the carbon stream formed 4.9 mM ccMA (Fig. S2). We selected the bacterial solution in the growth plateau period for plate coating. Compared with the original bacteria, the colony that was light white and transparent was MPDSΔsalC(pUCP18k-catA), while the opaque and slightly yellowish colony was B6-2ΔcatBΔsalC (Fig. S1). Based on the morphology and count of colony-forming units (CFUs), we found that MPDSΔsalC(pUCP18k-catA) gradually prevailed as the dominant strain during consortium growth (data not shown). However, competition between strains made it difficult to maintain a relatively stable and constant degradation curve. Therefore, consortia of resting cells were used to measure degradation of combined substrates and ccMA production with a wider range of timescales.

**Fig. 5:**
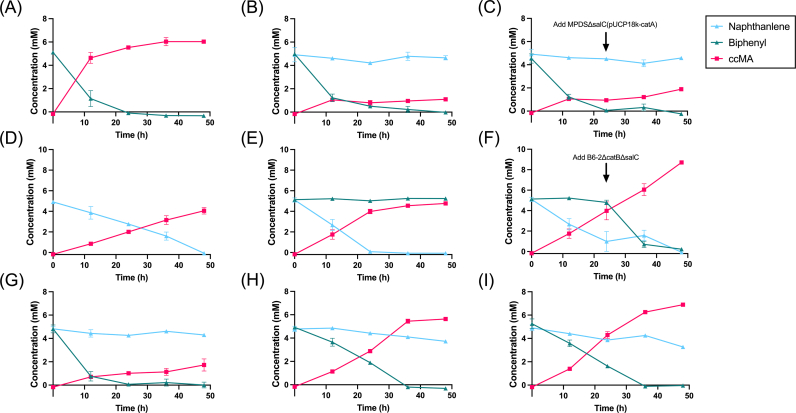
Mixed substrate degradation and ccMA accumulation in resting cells by microbial consortia. (A) B6-2ΔcatBΔsalC in biphenyl. (B) B6-2ΔcatBΔsalC in biphenyl and naphthalene. (C) B6-2ΔcatBΔsalC in biphenyl and naphthalene, MPDSΔsalC(pUCP18k-catA) was added after 24 h. (D) MPDSΔsalC(pUCP18k-catA) in naphthalene. (E) MPDSΔsalC(pUCP18k-catA) in biphenyl and naphthalene. (F) MPDSΔsalC(pUCP18k-catA) in biphenyl and naphthalene, B6-2ΔcatBΔsalC was added after 24 h. (G) Resting cells count ratio (B6-2ΔcatBΔsalC: MPDSΔsalC(pUCP18k-catA)) = 2: 1, in biphenyl and naphthalene. (H) Resting cells count ratio = 1: 1, in biphenyl and naphthalene. (I) Resting cells count ratio = 1: 2, in biphenyl and naphthalene. Symbols: filled squares (red), ccMA; filled triangles (green), biphenyl; filled triangles (blue), naphthalene. Initial concentration of each substrate is 5 mM. Each value shown is the means of the results of a triplicate experiment. Error bars indicate standard deviations.

**Fig. 6:**
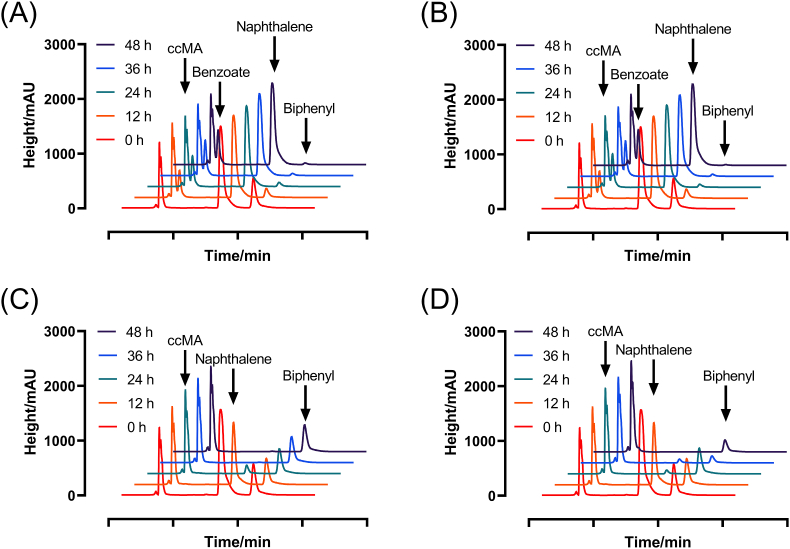
Chromatogram of compounds detected in resting cells of consortia. (A) B6-2ΔcatBΔsalC. (B) B6-2ΔcatBΔsalC and MPDSΔsalC(pUCP18k-catA) (added after 24 h). (C) MPDSΔsalC(pUCP18k-catA). (D) MPDSΔsalC(pUCP18k-catA) and B6-2ΔcatBΔsalC (added after 24 h). Symbols: red line, sampling at 0 h; orange line, sampling at 12 h; green line, sampling at 24 h; blue line, sampling at 36 h; black line, sampling at 48 h. The retention time of compounds: ccMA (2.92 min), benzoate (3.48 min), naphthalene (7.69 min) and biphenyl (10.25 min). The substrates of each chart are 5 mM biphenyl and 5 mM naphthalene. Chart A and B showed visible signals of benzoate accumulation.

Strains generating ccMA achieved essentially full conversion rates with a single substrate (Fig. 5A and D). In the presence of biphenyl and naphthalene, B6-2ΔcatBΔsalC only yielded about 1.09 mM ccMA and MPDSΔsalC(pUCP18k-catA) yielded 4.76 mM (Fig. 5B and E). As opposed to a solitary strain in a mixed substrate, another strain of the same biomass was added directly after 24 h. Subsequent addition of B6-2ΔcatBΔsalC led to the degradation of biphenyl and further accumulation of ccMA (Fig. 5F), while adding MPDSΔsalC(pUCP18k-catA) had no significant effect on degradation of naphthalene (Fig. 5C); sequential change in the addition of bacteria brought an increase in the molar conversion of consortia from 20% (B6-2ΔcatBΔsalC added first) to 85.2% (MPDSΔsalC(pUCP18k-catA) added first) (Table 2). Accompanied by B6-2ΔcatBΔsalC at the outset with different ratios, it was hard for MPDSΔsalC(pUCP18k-catA) to degrade naphthalene, especially when MPDSΔsalC(pUCP18k-catA) was not the major component of the artificial consortia (Fig. 5G–I). But the consortia with less B6-2ΔcatBΔsalC could degrade biphenyl and naphthalene and finally transform substrates to more than 5 mM ccMA. Compared with chromatograms of standard products, B6-2ΔcatBΔsalC accumulated benzoate once naphthalene was added, while MPDSΔsalC(pUCP18k-catA) added after 24 h was unable to effectively convert naphthalene to ccMA (Fig. 6).

**Table 2:** The conversion productivity [mg/(L·h)] and yield (mol/mol) of ccMA from biphenyl and naphthalene by double bacteria system.

Strains (with substrates biphenyl or naphthalene)	Conversion productivity and yield
B6-2ΔcatBΔsalC + MPDSΔsalC(pUCP18k-catA)	22.2, 58.0%
B6-2ΔcatBΔsalC: MPDSΔsalC(pUCP18k-catA) = 2: 1	5.1, 18.0%
B6-2ΔcatBΔsalC: MPDSΔsalC(pUCP18k-catA) = 1: 2	20.4, 67.8%
B6-2ΔcatBΔsalC + MPDSΔsalC(pUCP18k-catA) (added after 24 h)	5.6, 20.0%
MPDSΔsalC(pUCP18k-catA) + B6-2ΔcatBΔsalC (added after 24 h)	25.8, 85.2%

### 3.6 Fed-batch biological production of ccMA

**Fig. 7:**
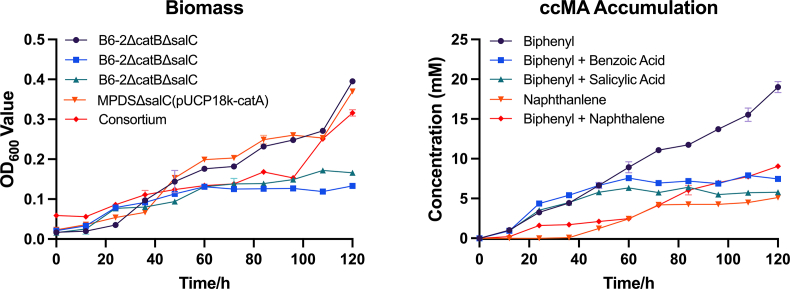
Fed-batch fermentation of strains using different carbon sources. (A) Growth curve of strains with different substrates. (B) ccMA production of strains with different substrates. Symbols: filled circles (black), B6-2ΔcatBΔsalC in biphenyl; filled squares (blue), B6-2ΔcatBΔsalC in biphenyl and benzoate; filled triangles (green), B6-2ΔcatBΔsalC in biphenyl and salicylate; filled inverted triangles (orange), MPDSΔsalC(pUCP18k-catA) in naphthalene; filled rhombuses (red), consortium in biphenyl and naphthalene. Each value shown is the means of the results of a triplicate experiment. Error bars indicate standard deviations.

Genetically modified strains B6-2, MPDS and consortium were cultivated in larger volumes to demonstrate the titer and mimic application scenarios (Fig. 7, S7). In culture of biphenyl, B6-2ΔcatBΔsalC produced 19.0 mM ccMA and hit OD_600nm_ of 0.4 after 120 h of cultivation. In co-culture substrates, either biphenyl-benzoate or biphenyl-salicylate could only support strain B6-2ΔcatBΔsalC growing to optical density less than 0.2 and plateaued at 60 h. Strains in these cultures could yield 7.5 mM ccMA from biphenyl-benzoate and 5.8 mM from biphenyl-salicylate. When naphthalene was utilized as the sole source, modified strain MPDS reached OD_600nm_ of 0.4 but only generated 5.1 mM ccMA. As previously mentioned, the consortia consisted of equal amount of B6-2ΔcatBΔsalC with MPDSΔsalC(pUCP18k-catA). In the presence of biphenyl and naphthalene, consortia produced 9.04 mM ccMA and had an OD_600_ of 0.32.

## 4 Discussion and conclusion

Synthesis of ccMA involves the shikimate and β-ketoadipate pathways. Most recent studies have focused on redesigning the shikimate pathway to further metabolize intermediates (e.g., chorismate) of food-based carbon sources to catechol, and ultimately to produce ccMA [11]. In the case of ccMA synthesis centered on the β-ketoadipic acid pathway, researchers have been more interested in converting lignin-derived aromatic hydrocarbons to ccMA [17,23]. We leveraged the central intermediate catechol from the upper pathway into the β-ketoadipate pathway, introducing petroleum-based aromatic molecules through a “biological funneling” approach [35]. In this study, the strategy considered the properties of the strains to degrade aromatic hydrocarbon pollutants and convert them into promising intermediates, such as muconate. The strategy allows for resource sustainability through the exploitation of lignin derivatives, and it is environmentally friendly and easy to conduct. The biphenyl and naphthalene of interest were classic representatives of PAH pollutants produced in industries. Additionally, the *bph* and *nah* operons in the upper pathway are metabolized to two key intermediates (benzoate and salicylate) during degradation. Therefore, benzoate and salicylate were also added. Apart from these, the feasibility of the “biological funnel” in upgrading a heterogeneous state was also verified by introducing a cluster of degradation genes for benzene and toluene.

While many strains degrade biphenyl, their efficiency is poor compared to selected strains *P. putida* B6-2 and *P. brassicacearum* MPDS. For example, *Brucella anthropi* MAPB-9 showed 66.15% degradation of biphenyl and still required 48 h for complete mineralization of biphenyl after the addition of glucose [36]. Strain B6-2 and its mutants grew on biphenyl as the sole carbon source with 100% degradation in 12 h. Comparing wild type to mutants, the declining OD_600_ value showed that knocked-out genes negatively affected strain growth. This phenomenon also appeared in strains cultivated with salicylate and benzoate. By focusing carbon flow on ccMA biosynthesis, strain B6-2ΔcatBΔsalC generated more ccMA than B6-2ΔcatB. When we applied this strategy to *Pseudomonas* sp. MPDS, the modified strain showed an identical ability to accumulate ccMA after IPTG induction and the final attainable biomass decreased with the degree of modification. The final conversion ratio of substrate to ccMA was nearly 1:1 for engineered strains of both B6-2 and MPDS, which indicated that GEMs constructed by this strategy could degrade target contaminants and convert them into high-value-added products.

Although modifying B6-2ΔcatBΔsalC did not impact the upper metabolic pathway of benzoate and salicylate, it reduced the ability to mineralize benzoate and salicylate. Expression of the *cat* genes in the lower pathway required only the metabolic intermediate ccMA as an inducer [37]. We inferred that the strain lacked the energy-producing step in the metabolism of both substances due to knockout of the *salC* gene. Co-metabolism is the process of microorganisms degrading a contaminant by energy- and carbon-acquiring enzymes during another compound's degradation [38]. In this study, co-metabolism was initiated by dioxygenase enzymes via converting biphenyl to dihydroxy-aromatic intermediates 2-hydroxypenta-2,4-dienoate. Therefore, biphenyl was used as an energy supporter for degrading benzoate and salicylate. The ccMA yield of B6-2ΔcatB was lower than that of B6-2ΔcatBΔsalC. By contrast, the double knockout strain exhibited a significant decrease in growth compared to the wild type and the single knockout. The presence of refractory substrates still had an inhibitory effect on the ability of the strain to degrade biphenyl. This circumstance also happened in *Sphingomonas* sp., whose biodegradation of fluorene is reduced by the presence of phenanthrene and fluoranthene [39].

Biological funneling to convert heterogeneous substrates to a single product was first developed for lignin-derived heterogeneous chemical mixtures [6]. By incorporating exogenous gene clusters, a funnel around the bio-valorization of complex aromatics was constructed with strain B6-2. Based on those results, the mutant B6-2ΔcatBΔsalC metabolized and converted benzene or toluene to ccMA with an exogenous catabolic cluster. This corresponded to a common way with an alternative host to expand catabolism capacity of more complex waste streams. But systematically characterizing genetic engineering may sequester the resources for host growth and finally lead to a depletion in growth rate [40]. In a consortium, researchers split a system into subcomponents and distribute cells into sub-populations specializing in individual chemical catabolism to balance burden and efficiency [41]. Compared with a single strain's performance in single or combined substrates, the ability of B6-2ΔcatBΔsalC to rapidly generate muconate was inhibited by the existence of naphthalene, which degraded biphenyl and metabolized only to benzoate and even further upstream.

Due to the weak ability of MPDSΔsalC(pUCP18k-catA) to degrade naphthalene in directly mixed consortia, we inferred that the operons of naphthalene degradation were suppressed by the organic acid (benzoic acid) intermediates produced by B6-2ΔcatBΔsalC. The premixed consortia inevitable confronted the degradation priority. The dominance of MPDSΔsalC(pUCP18k-catA) was because MPDSΔsalC(pUCP18k-catA) was not subject to growth inhibition by biphenyl, whereas B6-2 suffered to some extent from naphthalene. An increase in the proportion of MPDS during consortium metabolism correspondingly increased naphthalene degradation, elevating the terminal ccMA concentration, which was larger than the ccMA concentration achieved by full conversion of biphenyl. Interestingly, the combined substrate could be degraded and converted to ccMA when MPDS dominated the bulk in the pre- or long-term, which was shown in both the growth system and resting cells. Equivalently in this consortium, MPDSΔsalC(pUCP18k-catA) needed to degrade naphthalene beforehand, avoiding its inhibition by benzoic acid generated during biphenyl degradation and relieving the inhibition of B6-2ΔcatBΔsalC by naphthalene itself. Thus, this is a valid way to improve the breadth of a biological funneling platform via co-conversion.

This study revealed that *Pseudomonas* strains may utilize xenobiotics to catabolically accumulate ccMA. Based on the “biological funneling” theory, a genetic modification strategy was designed for the strains, and the breadth of funneling was expanded via co-metabolism and bacterial consortium. The results will broaden the applications of bio-funneling and contribute to the degradation and transformation of organic pollutants in the ambient environment.

## Declaration of competing interest

The authors declare that they have no competing interests.

## CRediT authorship contribution statement

**Siyang He:** Conceptualization, Methodology, Investigation, Writing – original draft, Writing – review & editing, All authors have read and agreed to the published version of the manuscript. **Weiwei Wang:** Conceptualization, Supervision, Methodology, Resources, Writing – review & editing, All authors have read and agreed to the published version of the manuscript. **Weidong Wang:** Conceptualization, Writing – review & editing, All authors have read and agreed to the published version of the manuscript. **Haiyang Hu:** Conceptualization, Writing – review & editing, All authors have read and agreed to the published version of the manuscript. **Ping Xu:** Conceptualization, Supervision, Resources, Writing – review & editing. **Hongzhi Tang:** Conceptualization, Supervision, Resources, Writing – review & editing, All authors have read and agreed to the published version of the manuscript.
