# Supplementary material for: Microbial production of cis,cis-muconic acid from aromatic compounds in engineered Pseudomonas
Source: Synth Syst Biotechnol. 2023 Aug 9;8(3):536–45. doi: 10.1016/j.synbio.2023.08.001 (PMC10448021; doi:10.1016/j.synbio.2023.08.001)
Supplement: Multimedia component 1 [file mmc1.pdf]

# Supplementary Information

**Microbial production of *cis,cis*-muconic acid from aromatic compounds in engineered *Pseudomonas***

Siyang He[a], Weiwei Wang[a]*, Weidong Wang[b], Haiyang Hu[a], Ping Xu[a], Hongzhi Tang[a]*

[a]State Key Laboratory of Microbial Metabolism, and School of Life Sciences & Biotechnology, Shanghai Jiao Tong University, Shanghai 200240, People's Republic of China

[b]College of life science, Northeast Forestry University, Harbin 150040, China

*Corresponding author: Dr. Weiwei Wang, Prof. Hongzhi Tang

Mailing address: State Key Laboratory of Microbial Metabolism, and School of Life Sciences & Biotechnology, Shanghai Jiao Tong University, Shanghai 200240, People's Republic of China

E-mail: oudigouzai@sjtu.edu.cn; tanghongzhi@sjtu.edu.cn.

Tel: +86-21-34204066; Fax: +86-21-34206723

## Running title

Microbial production of *cis,cis*-muconic acid by engineered *Pseudomonas*

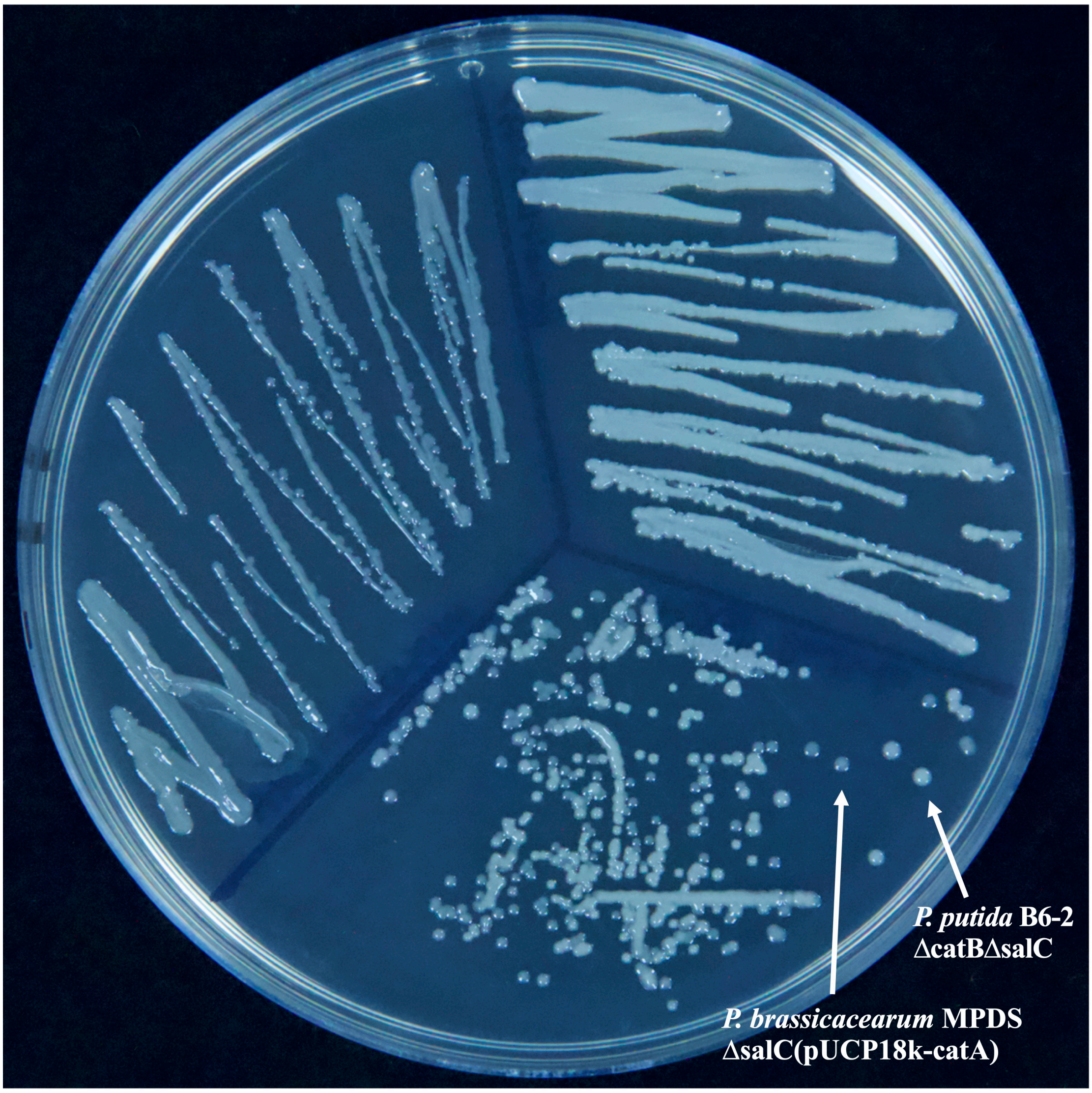


**Figure S1.** Colony morphology of strains in this study. Left upper: *P. brassicacearum* MPDSΔsalC(pUCP18k-catA); right upper: *P. putida* B6-2ΔcatBΔsalC; bottom: mixture of B6-2ΔcatBΔsalC and MPDSΔsalC(pUCP18k-catA).

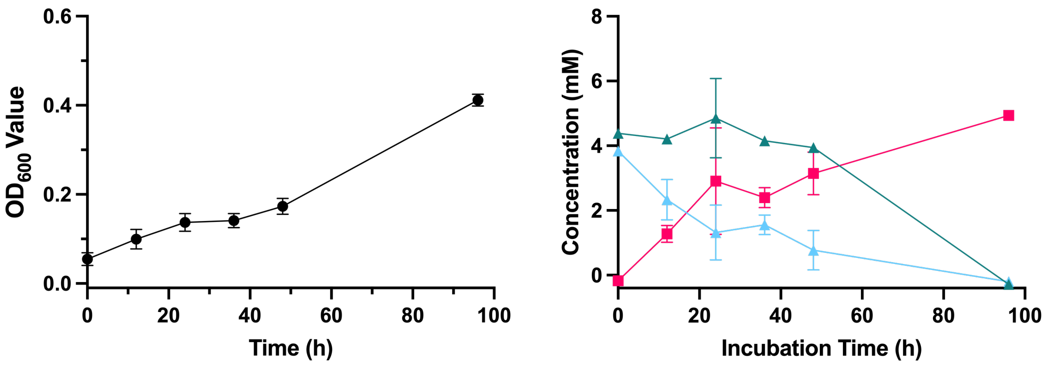


**Figure S2.** Optical density, substrates' degradation and ccMA accumulation of mixed bacteria. Left: optical density at 600 nm of the consortium (B6-2ΔcatBΔsalC and MPDSΔsalC(pUCP18k-catA)); right: concentration of biphenyl, naphthalene and ccMA. Symbols: filled squares (red), ccMA; filled triangles (green), biphenyl; filled triangles (blue), naphthalene. Each value shown is the means of the results of a triplicate experiment. Error bars indicate standard deviations.

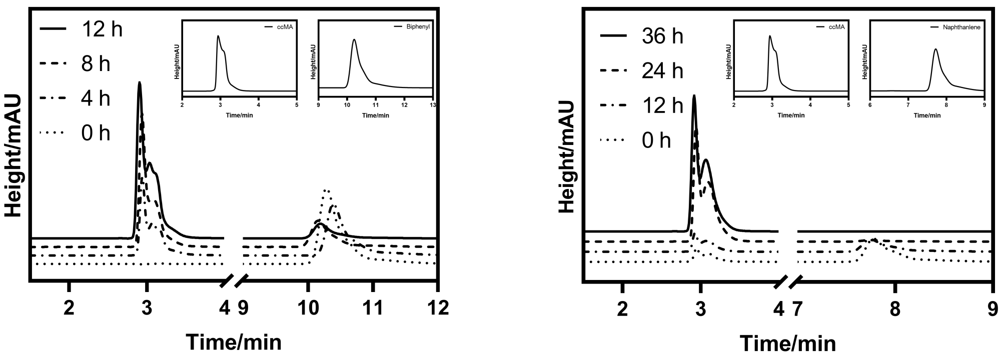


**Figure S3.** Chromatogram of biphenyl, naphthalene and ccMA. Left: *P. putida* B6-2ΔcatBΔsalC in MSM medium with 5 mM biphenyl; right: *P. brassicacearum* MPDSΔsalC(pUCP18k-catA) in MSM medium with 5 mM naphthalene.

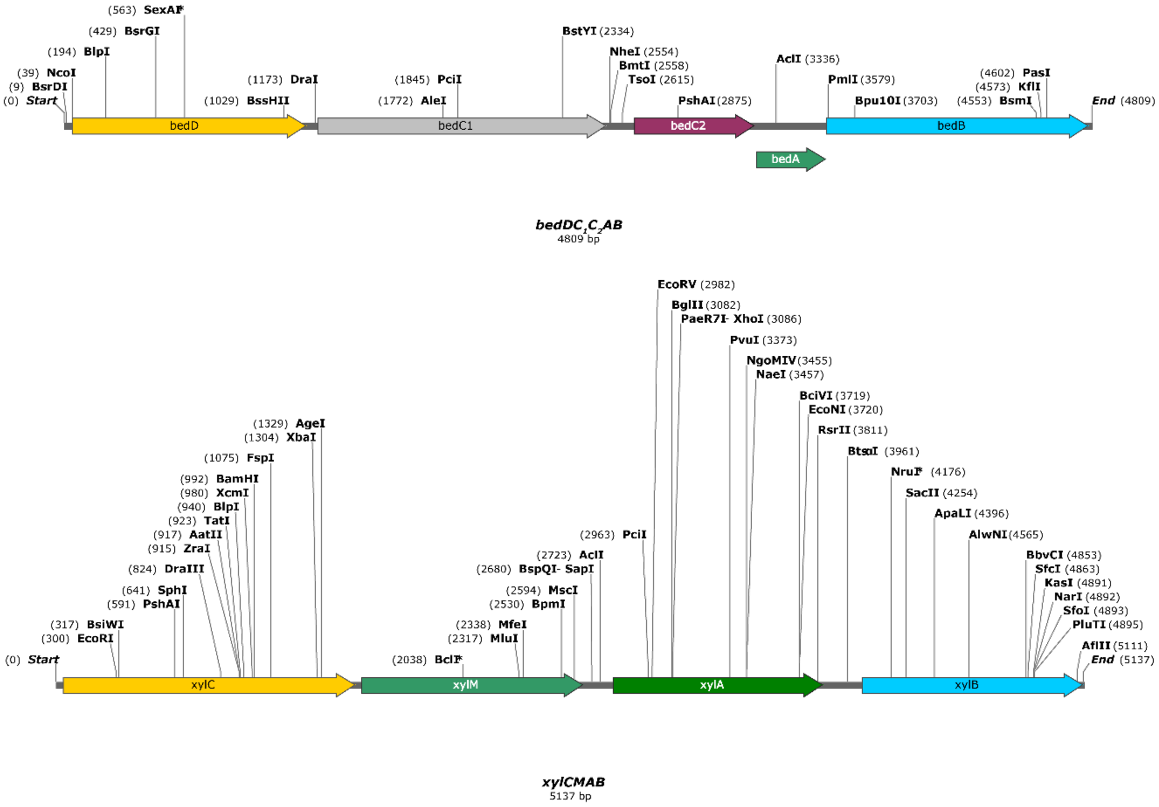


**Figure S4.** Map of *bedDC₁C₂AB* cluster and *xylCMAB* cluster

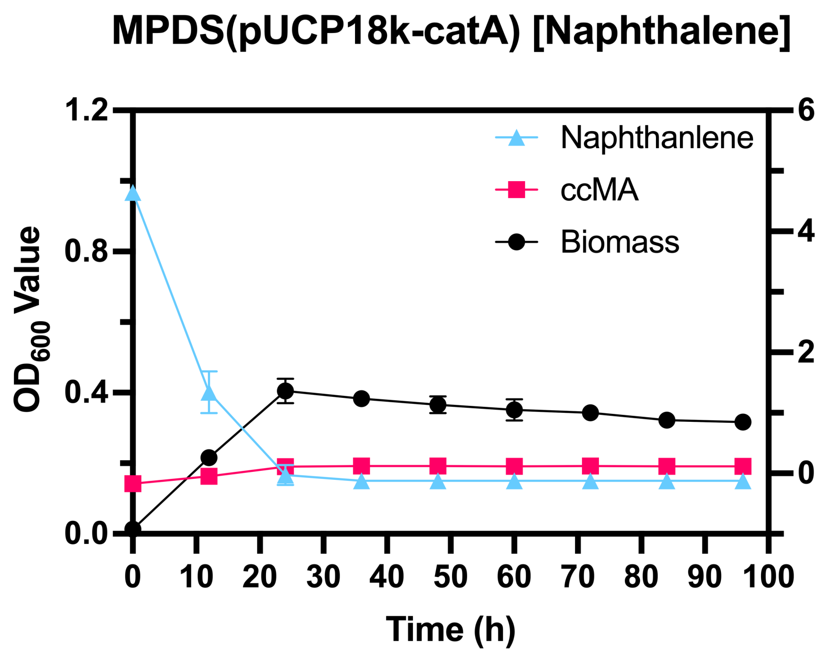


**Figure S5.** Growth of MPDS(pUCP18k-catA) and its ccMA accumulation using naphthalene. Each value shown is the means of the results of a triplicate experiment. Error bars indicate standard deviations.

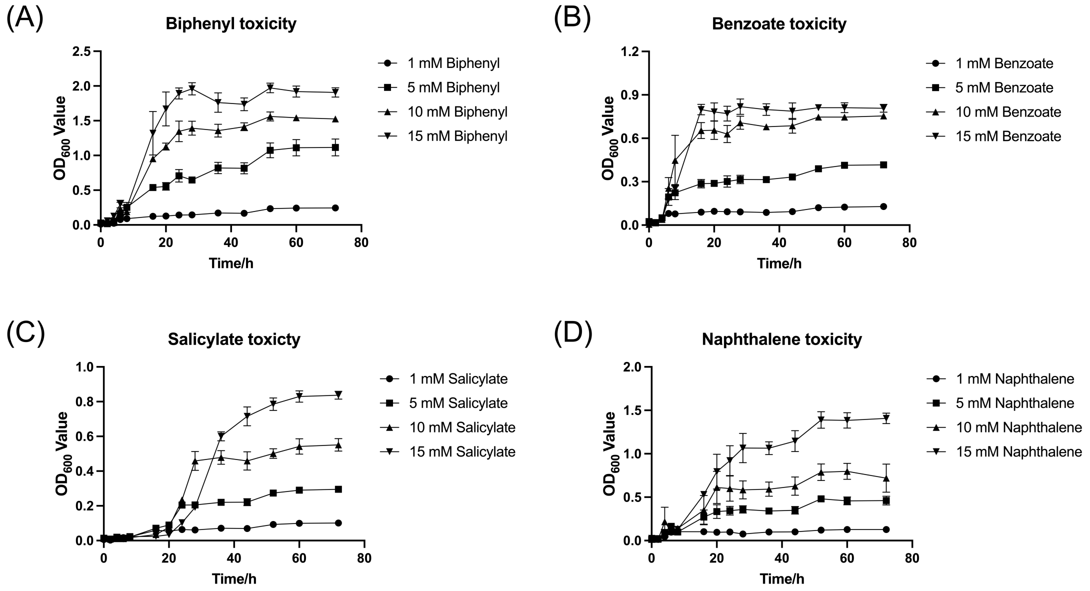


**Figure S6.** Growth curve of strains in different substances. (A-C) *P. putida* B6-2; (D) *P. brassicacearum* MPDS.

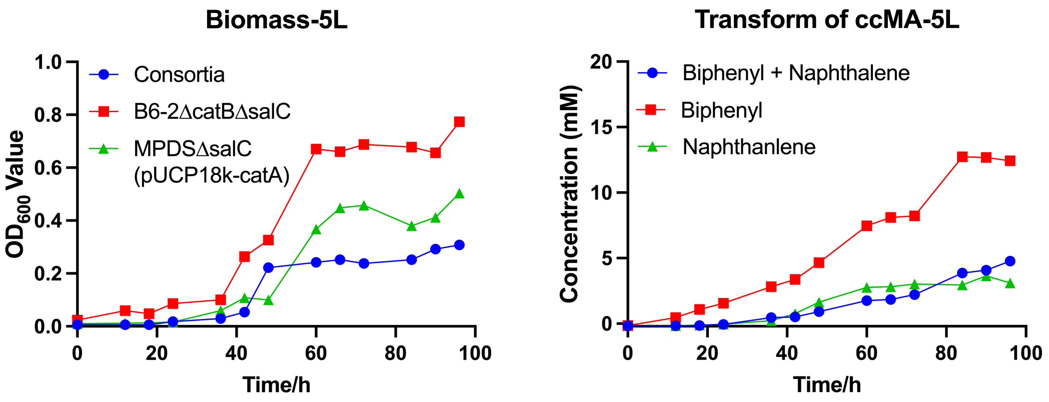


**Figure S7.** Fed-batch fermentation of strains in 5 L fermenter with different substrates.

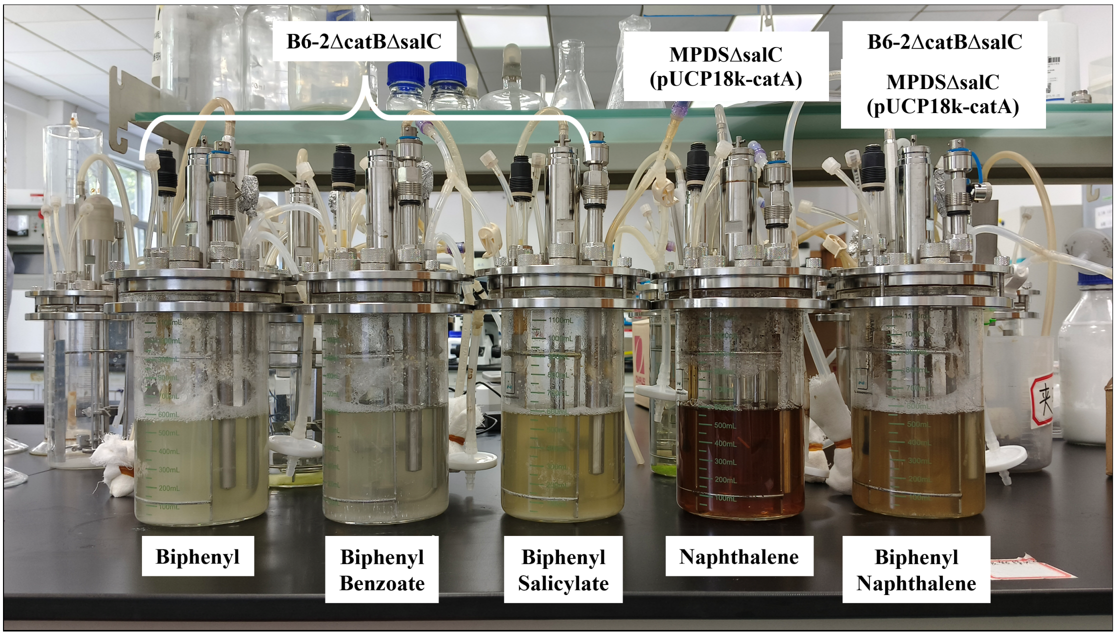


**Figure S8.** The 1 L fermenters for fed-batch fermentation after 120 h.

**Table S1.** Primers used in this study

| Primer | Sequence (5′→3′) |
|---|---|
| salC_A1 | GCATGAATTC CACTGACGCACATGTCG |
| salC_A2 | GTTATAAATTTGGAGTGTGA CATTATACCTTTGTTCATGAC |
| salC_B1 | TCACACTCCAAATTTATAAC GGTTCGGTTCGACTTATTGC |
| salC_B2 | AGCTGGATCC GCACGTTCTTGATCAGGTCG |
| catB_A1 | ATGCGGATCCATGACAAGCGCGCTGATTGAACAC |
| catB_A2 | AGCTAAGCTTGTTTGCCCTGGGCGTCGAGCAAGGC |
| catB_B1 | AGCTAAGCTTTGAGTCGATCGAAAGCGTCGAGGAC |
| catB_B2 | CAGCGAATTCATGGGGGATGTGCAGCTGGAAGTCG |
| pUCP18k(-catA)_rev | ACTGGCCGTCGTTTTACAACG |
| pUCP18k(-catA)_fwd | GGCGTAATCATGGTCATAGCTGT |
| catA(-pUCP18k)_rev | AAAACGACGGCCAGTTCAGCCCTCCTGCAACG |
| catA(-pUCP18k)_fwd | GACCATGATTACGCCATGACCGTGAAAATTTCCCACACT |

**Table S2.** Strains and plasmids used in this study

| Strain or plasmid | Description | Reference |
|---|---|---|
| **Strains** | | |
| *E. coli* DH5α TOP10 | General cloning host | Lab stock |
| *E. coli* S17-1 | An auxotroph of *E. coli* could transfer plasmid into *Pseudomonas* by conjugation | Lab stock |
| *P. putida* B6-2 | Wild-type strain | Lab stock |
| *P. brassicacearum* MPDS | Wild-type strain | Lab stock |
| B6-2ΔcatB | B6-2 derivative with knockout of *catB* | This study |
| B6-2ΔcatBΔsalC | B6-2 derivative with knockout of *catB* and *salC* | This study |
| MPDS(pUCP18k-catA) | MPDS derivative with insertion of *catA* | This study |
| MPDSΔsalC(pUCP18k) | MPDS derivative with knockout of *catB* and insertion of pUCP18k | |
| MPDSΔsalC(pUCP18k-catA) | MPDS derivative with knockout of *catB* and insertion of *catA* | This study |
| B6-2ΔcatBΔsalC(pUCP18k-xyl) | B6-2ΔcatBΔsalC derivative with insertion of *xylCMAB* | This study |
| B6-2ΔcatBΔsalC(pUCP18k-bed) | B6-2ΔcatBΔsalC derivative with insertion of $bedDC_1C_2AB$ | This study |
| **Plasmids** | | |
| pUCP18k | pUCP18 derivative, $Km^r$ | This study |
| pUCP18k-catA | pUCP18k derivative, *catA* inserted | This study |
| pUCP18k-bed | pUCP18k derivative, $bedDC_1C_2AB$ inserted | This study |
| pUCP18k-xyl | pUCP18k derivative, *xylCMAB* inserted | This study |
| pK18mob-SacB | Mobilizable vector, $mob^+$, *sacB*, $Km^r$ | This study |
| pK18-ΔcatB | pK18mobSacB derivative, Δ*catB* inserted | This study |
| pK18-ΔsalC | pK18mobSacB derivative, Δ*salC* inserted | This study |

**Table S3.** The synthesized DNA sequence used in this study

**Gene cluster *bed* of benzene upstream metabolism**

***bedD***

ATGGACCGTGCCATCCAGTCGCCCGGCAAGTACGTCCAGGGCGCCGACGC
CTTACAGCGTCTGGGCGACTACCTGAAGCCGCTGGCCGACTCGTGGCTGG
TGATCGCCGACAAGTTCGTCCTGGGCTTCGCCGAGGACACCATCCGTCAG
TCGCTGAGCAAGGCCGGCCTGGCGATGGACATCGTCGCCTTCAACGGCGA
GTGCTCGCAGGGCGAGGTGGACCGTCTGTGCCAGCTGGCCACCCAGAACG
GCCGTAGCGCCATCGTGGGCATCGGCGGCGGCAAGACCCTGGACACCGCC
AAGGCCGTCGCCTTCTTCCAGAAGGTCCCGGTCGCCGTCGCCCCGACCAT
CGCCTCGACCGACGCCCCGTGCAGCGCCCTGTCGGTGCTGTACACCGACG
AGGGCGAGTTCGACCGTTACCTGATGCTGCCGACCAACCCGGCCCTGGTG
GTGGTGGATACCGCCATCGTGGCCCGTGCCCCGGCCCGTCTGCTGGCCGC
CGGCATCGGCGACGCCCTGGCCACCTGGTTCGAGGCCCGTGCCGCCTCGC
GTAGCAGCGCCGCCACGATGGCCGGCGGCCCGGCCACCCAGACCGCCCTG
AACCTGGCCCGTTTCTGCTACGACACCCTGCTGGAGGAGGGCGAGAAGGC
CATGCTGGCCGTCCAGGCCCAGGTGGTGACCCCGGCCCTGGAGCGTATCG
TGGAGGCCAACACCTACCTGTCGGGCGTGGGCTTCGAGTCGGGCGGCGTC
GCCGCCGCCCACGCCGTGCACAACGGCCTGACCGCCGTGGCCGAGACCCA
CCACTTCTACCACGGCGAGAAGGTCGCCTTCGGCGTCCTGGTCCAGCTGG
CCCTGGAGAACGCCTCGAACGCCGAGATGCAGGAGGTCATGAGCCTGTGC
CACGCCGTGGGCCTGCCGATCACCCTGGCCCAGCTGGACATCACCGAGGA
CATCCCGACCAAGATGCGTGCCGTGGCCGAGCTGGCCTGCGCGCCGGGCG
AGACCATCCACAACATGCCGGGCGGCGTCACCGTGGAGCAGGTGTACGGC
GCCCTGCTGGTCGCCGACCAGCTGGGCCAGCACTTCCTGGAGTTCTGA

***bedC1***

ATGAACCAGACCGAGACCACCCCGATCCGTGTGCGCAAGAACTGGAAAA
CCTCGGAGATCGAGACCCTGTTCGACGAGCAGGCCGGCCGCATCGACCCG
CGTATCTACACCGACGAGGACCTGTACCAGCTGGAGCTGGAGCGTGTGTT
CGCCCGCTCGTGGCTGCTGCTGGGCCACGAGACCCACATCCGTAAGCCGG
GCGACTACTTCACCACCTACATGGGCGAGGACCCGGTGGTCGTGGTGCGT
CAGAAGGACGCCTCGATCGCCGTCTTCCTGAACCAGTGCCGTCACCGCGG
TATGCGTATCTGCCGTAGCGACGCCGGCAACGCCAAGGCCTTCACTTGCA
GCTACCACGGCTGGGCCTACGACACCGCCGGCAACCTGATCAACGTCCCC
TATGAGGCCGAGTCGTTCGCCTGCCTGGACAAGAAGGAGTGGAGCCCGCT
GAAGGCCCGTGTGGAGACCTACAAGGGCCTGATCTTCGCCAACTGGGACG
AGAACGCCATCGACCTGGACACCTACCTGGGCGAGGCCAAGTTCTACATG
GACCACATGCTGGACCGTACCGAGGCCGGCACCGAGGTGATCCCAGGCAT
CCAGAAGTGGGTCATCCCGTGCAACTGGAAGTTCGCCGCCGAGCAGTTCT
GCTCGGACATGTACCACGCCGGCACCACCGCCCACCTGTCGGGCATCATC
GCCGGCCTGCCGGAGGACCTGGAGCTGGCCGACCTGGCCCCGCCGAAGTT

CGGCAAGCAGTACCGTGCCTCGTGGGGCGGCCACGGCTCGGGCTTCTACA
TCGGCGACCCGAACATGATGCTGGCCATGATGGGCCCGAAGGTCACCAGC
TACCTGACCGAGGGCCCGGCCGCCGAGAAGGCCGCCGAGCGTCTGGGCTC
GATCGAGCGTGGCACCAAGATCATGCTGGAGCACATGACCGTCTTCCCGA
CGTGTAGCTTCCTGCCGGGCGTGAACACCATCCGTACCTGGCACCCGCGC
GGCCCGAACGAGGTGGAGGTGTGGGCCTTCACCGTGGTTGACGCCGACGC
CCCGGACGACATCAAGGAGGAGTTCCGTCGTCAGACCCTGCGCACCTTCA
GCGCCGGCGGCGTCTTCGAGCAGGACGACGGCGAGAACTGGGTCGAGAT
CCAGCACATCCTGCGCGGCCACAAGGCCCGCTCGCGTCCGTTCAACGCCG
AGATGTCGATGGGCCAGACCGTGGATAACGACCCGATCTACCCAGGCCGT
ATCAGCAACAACGTGTACTCGGAGGAGGCCGCCCGCGGCCTGTACGCCCA
CTGGCTGAAGATGATGACCAGCCCGGACTGGGAGGCCCTGAAGGCCACCC
GTTGA

***bedC2***

ATGATCGACTCGGTCAACCGTGCCGACCTGTTCCTGCGTAAGCCGGCCCC
GGTGGCCCTGGAGTTGCAAAACGAGATCGAGCAGTTCTACTACTGGGAGG
CCAAGCTGCTGAACGACCGTCGTTTCGACGAGTGGTTCGCCCTGCTGGCC
AAGGACATCCACTACTTCATGCCGATCCGCACCACCCGTATCATGCGTGA
CTCGCGTCTGGAGTACAGCGGCCTGCGTGACTACGCCCACTTCGACGACG
ACGCCACCATGATGAAGGGCCGCCTGCGCAAGATCACCTCGGACGTGTCG
TGGTCGGAGAACCCGGCCTCGCGTACCCGTCACATCGTGAGCAACGTGAT
GATCATCCCGACCGAGGTGGAGGGCGAGTACGAGATCAGCAGCACCTTCA
TCGTGTACCGTAACCGTCTGGAGCGCCAGCTGGACATCTTCGCCGGCGAG
CGCCGCGACCGTCTGCGTCGCAACAAGGGCGAGGCCGGCTTCGAGATCGT
CAACCGCACCATCCTGATCGACCAGAGCACCATCCTGGCCAACAACCTGT
CGTTCTTCTTCTGA

***bedB***

ATGACCTGGACCTACATCCTGCGCCAGTCGGACCTGCCGCCGGGCGAGAT
GCAGCGTTACGAGGGCGGCTCGGAGCCGGTCATGGTGTGCAACGTTGACG
GCGAGTTCTTCGCCGTCCAGGACACCTGCACCCACGGCGACTGGGCCCTG
TCGGAGGGCTACCTGGACGGCGACGTGGTGGAGTGCACCCTGCACTTCGG
CAAGTTCTGCGTGCGTACCGGCAAGGTGAAGGCCCTGCCGGCCTGCAAGC
CGATCAAGGTCTACCCGATCAAGATCGAGGGCGACGAGGTCCACGTAGAC
CTGGACAACGGCGAGCTGAAGTGA

***bedA***

ATGGCCAACCACGTGGCCATCATCGGCAACGGCGTCGCCGGCTTCACCAC
CGCCCAGGCCCTGCGTGCCGAGGGCTACGAGGGCCGTATCAGCCTGATCG
GCGAGGAGCAGCACCTGCCCTACGACCGTCCGAGCCTGAGCAAGGCCGTC
CTGGACGGCTCGTTCGAGCAGCCGCCGCGTCTGGCCGAGGCCGACTGGTA
CTCGGAGGCCTCGATCGAGATGCTGACCGGCTCGGAGGTGACCGACCTGG
ACACCCAGAAGAAGATGATCTCGCTGAACGACGGCAGCACCATCAGCGC

CGACGCCATCGTCATCGCCACCGGCTCGCGTGCCCGGATGCTGTCGCTGC
CGGGCTCGCAGCTGCCGGGCGTGGTCACCCTGCGTACCTACGGCGACGTC
CAGCTGCTGCGTGACTCGTGGACCCCGAACACCCGTCTGCTGATCGTGGG
CGGCGGCCTGATCGGCTGCGAGGTCGCCACCACCGCCCGTAAGCTGGGCC
TGTCGGTGACCATCCTGGAGGCCGGCGACGAGCTGCTGGTCCGTGTGCTG
GGCCGTCGTATCGGCGCCTGGCTGCGCGGCCTGCTGACCGAGCAGGGCGT
GCAGGTGGAGCTGAAAACCGGGGTGTCGGGCTTCTCGGGCGAGGGCCAG
CTGGAGAAGGTCATGGTCAACGACGGCCGTTCGTTCATCGCCGACAACGC
CCTGATCTGCGTGGGCGCCGACCCGGCCGACCAGCTGGCCCGTCAGGCCG
GCCTGGAGTGCGACCGCGGCGTGGTGGACCACCGCGGCGCCACCAGC
GCCAAGGGCATCTTCGCCGTGGGCGACGTCGCCACCTGGCCGCTGCACTC
GGGCGGCAAGCGTAGCCTGGAGACCTACATGAACGCCCAGCGTCAGGCC
ACCGCCGTCGCCAAGGCCATCCTGGGCAAGGAGGTGAGCGCCCCGCAGCT
GCCGGTGTCGTGGACCGAGATCGCCGGCCACCGAATGCAGATGGCCGGCG
ACATCGAGGGACCCGGCGAGTACGTCCTGCGCGGCACCCTGGGCATCGGC
AGCGCCCTGCTGTTCCGTCTGCTGGACGGCCGTATCCAGGCCGTGGTCGCC
GTGGACGCCCCGCGCGACTTCGCCCTGGCCAACCGTCTGGTCGAGGCCCA
GGTGATCATCGAGCCGGAGAAGCTGGCCGACGTGTCGAACAACATGCGTG
ACATCGTCCGTGCCAACGAGGGCAACCAGAAGTGA

**Gene cluster *xyl* of benzene upstream metabolism**

***xylC***

ATGCGTGAGACCAAGGAGCAGCCGATCTGGTACGGCAAGGTGTTCAGCTC
GAACTGGGTGGAGGCCCGCGGCGGCGTCGCCAACGTCGTCGATCCGAGCA
ACGGCGACATCCTGGGCATCACCGGCGTCGCCAACGGCGAGGACGTTGAT
GCCGCCGTGAACGCCGCCAAGCGTGCCCAGAAGGAGTGGGCCGCCATCCC
GTTCTCGGAGCGTGCCGCCATCGTCCGTAAGGCCGCCGAGAAGCTGAAGG
AGCGCGAGTACGAGTTCGCCGACTGGAACGTGCGTGAGTGCGGCGCCATC
CGTCCGAAGGGCCTGTGGGAGGCCGGCATCGCCTACGAGCAGATGCACCA
GGCCGCCGGCCTGGCCTCGCTGCCGAACGGCACCCTGTTCCCGAGCGCCG
TTCCCGGCCGTATGAACCTGTGCCAGCGTGTCCCGGTGGGCGTGGTGGGC
GTCATCGCCCCGTGGAACTTCCCGCTGTTCCTGGCCATGCGCTCGGTGGCC
CCGGCCCTGGCCCTGGGCAACGCCGTCATCCTGAAGCCGGACCTGCAAAC
CGCCGTGACCGGCGGCGCCCTGATCGCCGAGATTTTCTCGGACGCCGGTA
TGCCGGACGGCGTGCTGCACGTGCTGCCGGGCGGCGCCGACGTGGGCGAG
TCGATGGTCGCCAACTCGGGCATCAACATGATCTCGTTCACCGGCTCGAC
CCAGGTGGGCCGTCTGATCGGCGAGAAGTGCGGCCGTATGCTGAAGAAG
GTGGCCCTGGAGCTGGGCGGCAACAACGTCCACATCGTGCTGCCGGACGC
CGACCTGGAGGGCGCCGTCAGCTGCGCCGCCTGGGGCACCTTCCTGCACC
AGGGCCAGGTCTGCATGGCCGCCGGCCGTCACCTGGTCCACCGCGACGTC
GCCCAGCAGTACGCCGAGAAGCTGGCCCTGCGTGCCAAGAACCTGGTCGT
GGGCGACCCGAACAGCGACCAGGTCCACCTGGGCCCGCTGATCAACGAG
AAGCAGGTGGTCCGTGTCCACGCCCTGGTCGAGAGCGCCCAGCGTGCCGG
CGCCCAGGTGCTGGCCGGCGGCACCTACCAGGACCGCTACTACCAGGCCA

CCGTGATCATGGACGTCAAGCCGGAGATGGAGGTCTTCAAGTCGGAGATA
TTCGGCCCGGTCGCCCCGATCACCGTCTTCGACAGCATCGAGGAGGCCAT
CGAGCTGGCCAACTGCTCGGAGTACGGCCTGGCCGCCTCGATCCACACCC
GTGCCCTGGCCACCGGCCTGGACATCGCCAAGCGTCTGAACACCGGCATG
GTCCACATCAACGACCAGCCGATCAACTGCGAGCCGCACGTCCCGTTCGG
CGGCATGGGCGCCTCGGGCTCGGGCGGCCGTTTCGGCGGCCCGGCCTCGA
TCGAGGAGTTCACCCAGTCGCAGTGGATCAGCATGGTGGAGAAGCCGGCC
AACTACCCGTTCTGA

***xylM***

ATGGACACGCTTCGTTATTACCTGATTCCTGTTGTTACTGCTTGCGGGCTG
ATCGGATTTTACTATGGTGGCTATTGGGTTTGGCTTGGGGCGGCAACATTC
CCTGCACTGATGGTGCTTGATGTCATTTTACCGAAGGATTTTTCGGCCAGA
AAGGTAAGTCCCTTTTTCGCAGACCTTACCCAGTATTTGCAGTTACCATTA
ATGATCGGTCTATATGGGCTCCTTGTCTTCGGAGTTGAAAACGGGCGTATC
GAACTTAGTGAGCCGTTACAAGTGGCAGGGTGCATTCTTTCTTTGGCTTGG
CTTAGTGGTGTGCCAACTCTTCCGGTTTCGCATGAGTTGATGCATCGTCGC
CACTGGTTGCCTCGGAAAATGGCGCAGCTATTGGCTATGTTTTATGGTGAT
CCGAACCGAGACATTGCCCATGTCAACACGCATCACCTTTACTTAGATAC
GCCTCTCGATAGCGATACTCCGTACCGTGGTCAGACAATTTACAGTTTCGT
GATCAGTGCGACAGTTGGTTCCGTCAAAGATGCGATAAAGATTGAGGCTG
AAACTTTACGTAGAAAAGGACAGTCACCGTGGAATTTGTCCAACAAAACA
TATCAATATGTCGCACTTCTGCTCGCTCTGCCTGGCTTGGTTTCTTATCTGG
GCGGGCCAGCATTAGGGTTGGTTACGATTGCTTCGATGATTATTGCGAAA
GGGATAGTCGAGGGTTTTAATTACTTTCAGCACTATGGTTTAGTACGCGAT
TTAGATCAGCCTATCCTCCTGCACCACGCGTGGAATCATATGGGAACAAT
TGTGCGCCCGCTGGGTTGCGAAATTACTAACCATATCAATCATCATATTGA
CGGCTATACACGGTTCTATGAGTTGCGTCCGGAAAAAGAAGCCCCGCAGA
TGCCTTCGCTCTTTGTGTGTTTCCTTCTAGGGCTTATTCCGCCTCTTTGGTT
CGCTCTCATTGCAAAACCAAAGTTGAGAGACTGGGACCAGCGGTACGCAA
CTCCAGGTGAGCGCGAACTGGCTATGGCTGCAAATAAAAAAGCGGGATG
GCCACTGTGGTGTGAAAGTGAACTGGGTCGGGTGGCTAGCATTTGA

***xylA***

ATGAATGAGTTTTTTAAGAAAATCTCTGGTTTATTTGTGCCGCCTCCGGAA
TCTACCGTTTCAGTCAGAGGGCAGGGGTTTCAGTTTAAGGTGCCACGCGG
GCAAACCATTCTGGAAAGCGCTCTGCATCAAGGAATTGCCTTTCCGCATG
ATTGCAAAGTCGGATCTTGTGGGACATGTAAATATAAACTGATATCTGGC
AGGGTCAATGAGTTGACCTCTTCTGCTATGGGTCTGAGTGGCGATCTGTAT
CAGTCCGGCTATCGTTTGGGTTGTCAATGCATACCAAAAGAAGATCTCGA
GATAGAGCTAGACACAGTGCTCGGGCAGGCGTTAGTTCCAATAGAAACGA
GTGCCTTGATTAGTAAGCAGAAACGGCTGGCGCACGATATAGTCGAGATG
GAAGTAGTGCCCGATAAGCAGATAGCCTTCTACCCCGGCCAGTATGCAGA
TGTAGAATGTGCAGAATGCTCTGCTGTAAGGAGTTATTCTTTTTCCGCTCC

GCCCCAACCTGACGGCTCCCTGAGCTTCCATGTTCGCCTTGTCCCAGGTGG
AGTTTTCAGTGGTTGGCTATTTGGTGGCGATCGTACAGGAGCGACACTAA
CCCTGCGAGCGCCTTATGGACAGTTCGGGCTCCATGAGAGCAATGCCACG
ATGGTCTGCGTAGCCGGCGGAACGGGGCTTGCTCCAATTAAATGTGTTTT
GCAGAGCATGACCCAGGCCCAGCGAGAGCGTGATGTGTTGTTGTTCTTTG
GAGCTCGTCAACAACGTGACCTATATTGCCTCGACGAAATAGAAGCGCTG
CAACTCGATTGGGGTGGGCGCTTCGAGCTTATTCCAGTTTTGTCCGAAGAG
TCTTCTACGTCGTCATGGAAAGGGAAACGTGGCATGGTAACCGAGTATTT
TAAGGAGTACCTCACTGGGCAGCCTTATGAAGGATACCTTTGCGGGCCGC
CCCCTATGGTGGACGCTGCCGAGACCGAGCTCGTTCGACTTGGTGTTGCG
CGGGAATTAGTGTTTGCGGACCGTTTTTATAATAGACCTCCTTGCTAG

***xylB***

ATGGAAATCAAAGCAGCAATAGTTCGCCAAAAAAATGGCCCGTTCTTACT
TGAGCATGTAGCTCTTAATGAGCCAGCTGAAGATCAGGTTCTCGTTAGATT
GGTTGCAACCGGGCTGTGTCATACGGATCTGGTTTGTCGCGATCAGCATTA
TCCGGTTCCACTACCGATGGTATTTGGGCATGAAGGGGCTGGTGTGGTTG
AGCGGGTTGGGTCCGCGGTCAAAAAGGTTCAGCCGGGCGACCATGTTGTT
TTGACATTTTATACCTGCGGGAGTTGTGATGCTTGTCTTTCCGGAGACCCT
ACCAGTTGTGCAAACTCATTTGGCCCTAACTTTATGGGGCGCTCGGTAACC
GGGGAGTGCACCATCCACGATCACCAAGGGGCAGAGGTGGGAGCAAGCT
TTTTTGGGCAGTCCTCCTTTGCGACATATGCGCTATCTTATGAACGTAACA
CTGTGAAGGTTACAAAAGACGTACCGCTTGAGTTGCTTGGGCCTCTTGGTT
GTGGCATTCAAACTGGCGCAGGGTCTGTTCTGAATGCGCTTAATCCGCCA
GCGGGTTCTGCTATCGCAATTTTTGGTGCTGGGGCAGTTGGTCTTTCGGCC
GTGATGGCTGCCGTTGTAGCAGGTTGTACCACCATCATCGCTGTCGACGTT
AAGGAAAACCGGCTGGAACTAGCCAGTGAACTTGGGGCGACGCACATTA
TTAACCCGGCCGCTAACGATCCCATTGAGGCGATCAAAGAGATATTCGCT
GACGGTGTTCCGTATGTATTGGAGACTAGCGGTTTGCCCGCCGTGCTTACG
CAGGCGATCCTCAGCTCTGCTATAGGCGGTGAGATCGGTATTGTAGGGGC
GCCACCTATGGGGGCCACGGTGCCCGTTGACATTAACTTCCTGCTATTCAA
TCGTAAGCTTCGTGGAATCGTTGAGGGTCAGTCGATCTCGGATATTTTCAT
TCCCAGGCTGGTGGAGCTTTATCGCCAGGGGAAGTTTCCGTTTGACAAGC
TGATTAAGTTTTATCCTTTTGATGAAATCAATCGAGCCGCCGAAGATTCGG
AAAAAGGCGTGACGCTTAAGCCGGTACTCCGGATTGGTTGA
